# Targeting ATR Pathway in Solid Tumors: Evidence of Improving Therapeutic Outcomes

**DOI:** 10.3390/ijms25052767

**Published:** 2024-02-27

**Authors:** Dimitra Mavroeidi, Anastasia Georganta, Emmanouil Panagiotou, Konstantinos Syrigos, Vassilis L. Souliotis

**Affiliations:** 1 Institute of Chemical Biology, National Hellenic Research Foundation, 116 35 Athens, Greece; dmavro@eie.gr; 2 Third Department of Medicine, Sotiria General Hospital for Chest Diseases, National and Kapodistrian University of Athens, 115 27 Athens, Greece; anastasiagew107@gmail.com (A.G.); empanagi@med.uoa.gr (E.P.); ksyrigos@med.uoa.gr (K.S.)

**Keywords:** ATR, ATR inhibitor, ceralasertib (AZD6738), DNA damage response, immune system, synthetic lethality, ATR-ATM interplay

## Abstract

The DNA damage response (DDR) system is a complicated network of signaling pathways that detects and repairs DNA damage or induces apoptosis. Critical regulators of the DDR network include the DNA damage kinases ataxia telangiectasia mutated Rad3-related kinase (ATR) and ataxia-telangiectasia mutated (ATM). The ATR pathway coordinates processes such as replication stress response, stabilization of replication forks, cell cycle arrest, and DNA repair. ATR inhibition disrupts these functions, causing a reduction of DNA repair, accumulation of DNA damage, replication fork collapse, inappropriate mitotic entry, and mitotic catastrophe. Recent data have shown that the inhibition of ATR can lead to synthetic lethality in ATM-deficient malignancies. In addition, ATR inhibition plays a significant role in the activation of the immune system by increasing the tumor mutational burden and neoantigen load as well as by triggering the accumulation of cytosolic DNA and subsequently inducing the cGAS-STING pathway and the type I IFN response. Taken together, we review stimulating data showing that ATR kinase inhibition can alter the DDR network, the immune system, and their interplay and, therefore, potentially provide a novel strategy to improve the efficacy of antitumor therapy, using ATR inhibitors as monotherapy or in combination with genotoxic drugs and/or immunomodulators.

## 1. Introduction

Cells face constant exposure to multiple DNA damage sources, both endogenous (e.g., oxidation, alkylation, hydrolysis, mismatch of DNA bases) and exogenous (genotoxic chemicals, UV light, ionizing radiation, etc.) [1,2,3,4,5]. To neutralize these threats and ensure genomic stability, cells have developed several mechanisms, collectively called the DNA damage response (DDR) network [6]. The DDR system includes damage sensors, transducer kinases, and effectors to maintain genomic integrity. Interestingly, recent data have shown that the deregulated DDR network is capable of activating the host immune system [7]. These results potentially provide a novel strategy for enhancing the efficacy of immunotherapy.

On the other hand, deregulated DDR pathways trigger mutagenesis and genomic instability, thus getting implicated in the onset and progression of cancer. Cancer cells divide continuously due to a breakdown of the mechanisms regulating the cell cycle. The increased proliferation rate and the DNA repair defects in cancer cells make these cells more vulnerable to specific DDR inhibition [8]. Hence, DDR inhibitors, a class of drugs that can modify the DDR network, have recently gained great attention in cancer treatment research. The known DDR inhibitors include drugs that inhibit several DNA repair pathways or molecular components, such as the polyADP-ribose polymerase (PARP), the ataxia telangiectasia mutated kinase (ATM), the ataxia telangiectasia and Rad3-related kinase (ATR), the Checkpoint kinases 1 and 2 (CHK1/2), the Cyclin-dependent kinases 4 and 6, (CDK4/6), the cell-cycle checkpoint kinase WEE1, and the DNA-dependent protein kinase (DNA-PK) [8].

Particularly, ATM and ATR kinases have a critical role in the activation of the DDR network. As for ATR, following the formation of the stable replication protein A (RPA)-single-stranded DNA (ssDNA) complex at DNA damage sites, the ATR-interacting protein (ATRIP) binds to RPA, causing the localization of the ATR kinase to these sites [9]. Next, to give more time for the DNA repair mechanism to proceed, the ATR-CHK1 signaling pathway induces cell-cycle arrest at the G2-M phase. As for ATM, this kinase is activated via the MRN (meiotic recombination protein 11—MRE11, Nijmegen breakage syndrome protein 1—NBS1) complex, a DNA double-strand breaks (DSBs) sensor [10]. Then, ATM phosphorylates the H2A histone family member X at S139 (γH2AX) and induces the CHK2 kinase, resulting in G1-S and intra-S-phase checkpoint activation. Based on the above, ATR and ATM kinases may be promising molecular targets in the treatment of cancer. Currently, several ATM/ATR inhibitors have been identified and are participating in preclinical and clinical evaluation.

Herein, we present a review of the current literature summarizing the role of ATR inhibition in the modification of the DDR network, the immune system, and their interplay. The latest advances in ATR inhibitors in preclinical and clinical states are also elucidated.

## 2. The ATR Pathway in the DNA Damage Response Network

The DNA damage response network is activated following the detection of DNA damage by specific sensors [6]. The next step is the activation of a signal transduction cascade, which leads to the induction of genome protection mechanisms, such as DNA repair pathways, cell cycle checkpoints, or the initiation of apoptosis. Deregulated DDR may also result in mutagenesis and genomic instability. Since DDR is an important cellular network of molecular pathways that regulates the cell’s decision to either remove the DNA damage or to undergo cell death, it is implicated in both the pathogenesis and progression of a disease, as well as in the outcome of therapeutic treatment.

There are several DNA repair mechanisms active throughout the cell cycle. These include the Fanconi Anemia (FA) pathway, which is implicated in the repair of interstrand crosslinks (ICLs), the Nucleotide Excision Repair (NER), which removes adducts that disrupt the DNA double-helix, the Base Excision Repair (BER), coping with alkylated, oxidized and deaminated bases and the Mismatch Repair (MMR) pathway that resolves mismatched bases that may occur during DNA replication. In addition, the homologous recombination (HR) repair and the non-homologous DNA end joining (NHEJ) are two major subpathways for the repair of DSB, the most lethal type of DNA lesion [11]. Finally, the direct repair mechanism is a simple and accurate repair mechanism that removes alkyl groups from the O6 position of guanine to the cysteine residue of the O6-methylguanine-DNA methyltransferase (MGMT) [12].

**Figure 1:**
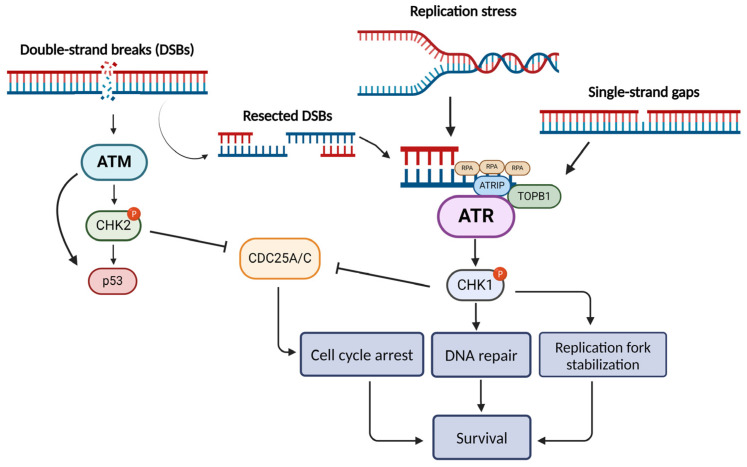
Schematic overview of the ATR/ATM pathways (Figure was created with BioRender.com).

It is generally accepted that in cells with dysfunctional DDR, such as cancer cells, DNA integrity is often compromised. During the S phase, the replication fork is usually stalled by DNA lesions, and if these remain unresolved, the replication machinery eventually collapses [13]. This condition is referred to as “replication stress” and is a common characteristic of tumor cells due to chronic proliferation, being also the main cause of genomic instability in cancer. Nonetheless, it may also be noticed in normal cells on account of oxidative stress or other endogenous damage [13]. DDR also needs to modulate cell cycle progression, as cell cycle arrest is required for the resolution of DNA lesions. Two major kinases appear to be the key players organizing the response right after DNA damage recognition: ATM and ATR (Figure 1) [14].

ATM is mostly activated in response to DSBs during all phases of the cell cycle [6,15], while ATR is involved in the recognition of single-strand breaks (SSBs), occurring as a response to numerous mechanisms (e.g., during replication fork stalling or as NER and DSBs repair intermediates) [14,16,17,18]. The broad involvement of ATR in various processes (replication stress response, SSBs and DSBs repair, interstrand crosslink repair, meiosis) is highlighted by the fact that ATR, and not ATM, is indispensable for cell survival [19,20]. Particularly, ATR is an essential protein with scarce loss-of-function mutations in cancer [21], while it has been observed that impaired ATR function in mouse models leads to tumorigenesis resistance [22].

Of note, previous studies have shown crosstalk between the ATR and the ATM pathways [23,24]. As far as DSB repair is concerned, ATM-dependent activation of ATR has been shown to occur [25,26]. DSBs are primarily detected by the MRN complex, which is vital for the activation of ATM [27,28,29,30,31]. During the DSB repair that is mediated by ATM signaling, ssDNA fragments are often accumulated as a result of the resection of DSBs by exo- and endo-nucleases [25]. These ssDNA fragments stimulate the ATR pathway, forming the ATR-ATM interplay during DSB repair [24].

Recent data have shown that in metazoan cells, stimulation of ATR kinase activity depends on two pathways: the DNA topoisomerase 2-binding protein 1 (TopBP1) and the Ewing tumor-associated antigen 1 (ETAA1) pathway. Whether the activation of ATR occurs by TOPBP1 or ETAA1 depends on the existence of DNA damage and the phase of the cell cycle [32]. ATR pathway initiates with the RPA protein recognizing and coating ssDNA, followed by the binding of ATR-interacting protein (ATRIP) and the assembly of ATR-ATRIP complex at the DNA damage sites [33]. Several additional regulatory proteins, such as the Rad17 complex, the Rad9–Rad1–Hus1 (9-1-1) complex, and the 9-1-1 interacting nuclear orphan (RHINO), need to be involved [24,34,35] in order to recruit the TOPBP1 that finally stimulates the kinase activity of ATR [20,24,35]. As for the ETAA1-mediated activation of ATR, ETAA1 interacts with RPA and activates ATR by the ATR activation domains [36]. Interestingly, ETAA1 regulates the transitions of the cell cycle even in the absence of replication stress or DNA lesions [37]. Consequently, with the aid of mediators such as claspin [38], ATR phosphorylates the downstream Checkpoint Kinase 1 (CHK1). This pathway can result in cell cycle arrest either in the intra-S-phase or in the G2/M phase [13,14], as CHK1 is responsible for the phosphorylation of multiple substrates, including phosphatases CDC25A, CDC25B, and CDC25C [20]. This results in either ubiquitin-related degradation or translocation far from their substrates and eventually prevents them from keeping the kinases CDK2 and CDK1 active, thus blocking cell cycle progression [39,40].

## 3. The ATR Pathway and the Interplay between the DDR Network and the Immune System

The Immune System and the DDR network are important mechanisms that are implicated in the survival of living organisms. Interestingly, a series of recent studies have shown that these two systems play a crucial role in the onset and progression of cancer, as well as in the outcome of anticancer therapy [41]. Traditionally, conventional chemotherapy was associated with immunosuppression, and many chemotherapeutics are applied to treat autoimmune diseases. On the other hand, a growing body of research indicates that agents that damage DNA can stimulate immunity in a number of ways, some of which may be useful for immunotherapy. Several mechanisms are implicated in the DDR-mediated activation of the immune system, including the following:

- The induction of immunogenic cell death (ICD), i.e., cell death, which elicits an immune response [42]. Not all modes of cell death induce such a response, which requires, in addition to neoantigen exposure, the presence of additional danger signals [43]. Such signals are provided by damage-associated molecular patterns (DAMPs), which are molecules released from dying tumor cells. DAMPs cause antigen-presenting cells to be drawn to the site, where they process and present tumor neoantigens, triggering an adaptive immune response. DAMPs released during chemotherapy-induced immunogenic cell death include, among others, DNA release in the cytoplasm, where it leads to activation of stimulator-of-interferon genes (STING) and induction of type I interferon (IFN) and pro-inflammatory cytokines [44].
- The increase in antigen presentation through the upregulation of MHC-1 (major histocompatibility complex type 1) expression on tumor cells and promotion of dendritic cell (DC) maturation is an innate response that leads to adaptive immunity [45].
- Changes in the cytokine milieu within the tumor microenvironment through the release of proinflammatory cytokines, including TNF-a, IL-6, and IFN-γ [46,47,48], have a direct effect on neighboring cells, resulting in an immunogenic tumor microenvironment [49].
- Downregulation of myeloid-derived suppressor cells (MDSC) and regulatory T-cells (Tregs), which play a role in dampening the host immune response [50,51].
- Modification of the expression of the immune checkpoint factors PD-1/PD-L1. Indeed, previous reports have shown genotoxic chemotherapy results in downregulation of the expression of PD-L1 [52] or a redistribution of this surface-expressed ligand to the nuclear membrane [53].
- Increase of the tumor neoantigen burden. There are indications that genotoxic drugs may enhance tumor immunogenicity by causing, thanks to their mutagenicity, an increase in tumor neoantigens, which appear to play a critical role in the effectiveness of immune checkpoint blockade immunotherapy [54,55,56].

Interestingly, previous studies have shown that a shift in the balance between DNA damage and repair results in the accumulation of cytosolic DNA that can act as a potent immune stimulator via the activation of the cGAS/STING pathway and the subsequent activation of the type-I interferon (IFN) signaling pathway [57,58,59]. Other studies have also shown that the progression of the cell cycle through mitosis in the presence of DNA DSBs results in the generation of micronuclei and the activation of the immune system [60,61]. Foreign DNA detection is a crucial step in the induction of immunity in many organisms. In mammalian cells, activation of the immune responses is contributed mainly by the cyclic GMP-AMP synthase (cGAS) –STING pathway, which plays an important role in coupling the detection of the DNA to the activation of the innate immune defense mechanisms [62]. Indeed, the binding of dsDNA to cGAS triggers its catalytic activity and results in the formation of 2′,3′-cyclic GMP–AMP (cGAMP), which acts as a potent agonist of STING [63,64]. The synthesis of cGAMP is an important step that results in the activation of the cGAS-mediated antiviral effects in several species [65]. Indeed, the cGAS molecule is activated by bacterial and viral DNA as well as by mitochondrial DNA and phagocytosed DNA that are abnormally localized in the cytosol. The induction of cGAS produces cGAMP that activates STING and leads to the induction of TANK-binding kinase 1 (TBK1), IkB kinase (IKK), and NF-kB-inducing kinase (NIK) [66,67]. Together, induction of these kinases triggers activation and nuclear transportation of IFN regulatory factor 3 (IRF3) and NF-kB, resulting in the expression of type I IFN, interferon-stimulated genes (ISGs) and inflammatory cytokines-further connecting the DDR network with the immune system [68,69]. On the other hand, extensive observations suggest that chronic activation of the cGAS/STING pathway can induce an immune suppression in the tumor microenvironment that promotes the progression of the tumor [70,71,72]. In line with these data, activation of the cGAS/STING pathway may have either a pro-tumor or an anti-tumor effect, depending on the stage of tumor progression and the cancer type.

Since cytoplasmic dsDNA can activate STING, chemotherapies that result in the accumulation of cytoplasmic dsDNA could be an interesting strategy for STING activation. Indeed, genotoxic therapies, including radiotherapy, cytotoxic chemotherapy, inhibitors of PARP and/or ATR, augmented cytosolic DNA damage-induced dsDNA and activate the cGAS-STING-IFN response [73,74,75,76] with S-phase DNA damage being a particularly potent activator [77]. The activation of cGAS/STING inflammatory responses following PARP [78] or ATR [79] inhibition may also induce the formation of micronuclei capable of activating innate immune responses [58,80]. Micronuclei are small organelles that contain DNA and are produced in the telophase of mitosis as a result of several genotoxic stressors [81]. Although these organelles are formed with a nuclear envelope (NE), after mitosis, they lose compartmentalization as their NE ruptures [82]. A critical result of micronuclei rupture is that chromosomal DNA becomes available to cGAS and leads to the activation of immune responses [58,60,61,83,84,85].

**Figure 2:**
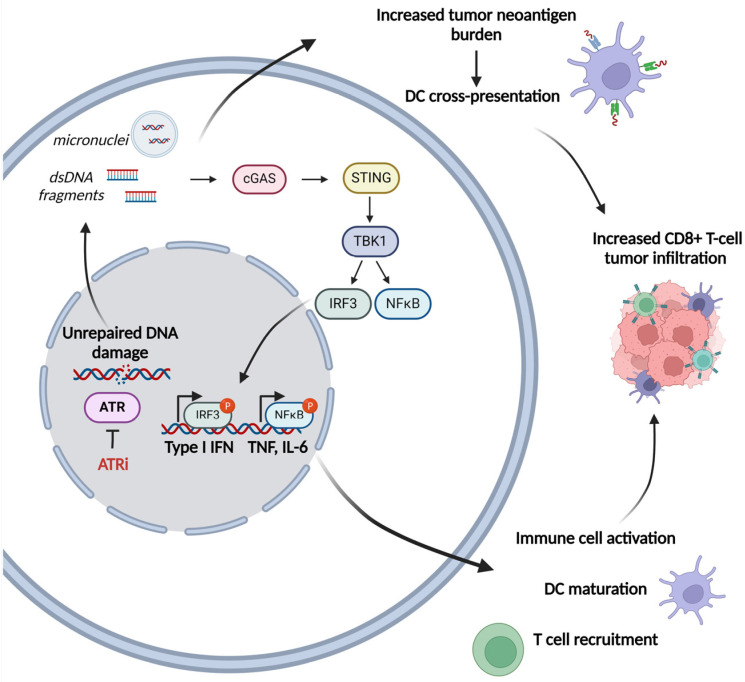
ATR pathway implication in the antitumor immunity (Figure was created with BioRender.com).

Concerning ATR inhibition, an accumulating body of evidence suggests that the ATR pathway modulates antitumor immunity (Figure 2). Indeed, ATR is induced by replication stress, single-stranded DNA, and increased R-loops, and activates a signaling cascade that involves CHK1 and WEE1 kinases that, in turn, leads to the induction of a cell cycle checkpoint in order to give more time to the DNA repair mechanism to remove lesions [86]. In line with these data, the inhibition of ATR leads to the disruption of these functions, resulting in inappropriate mitotic entry and mitotic catastrophe. Moreover, the cytosolic DNA thus released may induce cGAS-STING signaling and a type I interferon response. In addition, previous studies have investigated the role of ATR inhibition as a means of increasing the tumor mutational burden and the production of neoantigens, which may improve the sensitivity to immune checkpoint inhibitors due to elevated antigen presentation. That is, an analysis of data from The Cancer Genome Atlas and The Cancer Immunome Atlas demonstrated that samples with mutations in DDR-related genes, such as ATR, showed increased neoantigen levels [87], thus strengthening the rationale for combination therapies using PD-1/PD-L1 blocking and ATR inhibitors. This is supported by preliminary data in a syngeneic mouse model of head and neck squamous cell carcinoma (HNSCC), where ATR inhibition by AZD6738 resulted in cGAS/STING pathway activation and induced tumor infiltration of cytotoxic T cells that eventually achieved tumor growth arrest and prolonged survival [88].

In line with these results, previous studies have shown that inhibitors of ATR potentiate immune stimulation following exposure to radiotherapy. Indeed, combined treatment with radiotherapy and ATR inhibitor-induced type I/II IFN signaling and infiltration of CD8+ T-cells in a manner dependent on cGAS/STING [89,90,91]. While ATR inhibitors do not directly induce DNA damage, one may assume that the higher immunogenicity observed in irradiated tumors following ATR inhibition is due to the overriding of the G2/M cell cycle checkpoint. As a result, an elevated proportion of cells with non-repaired DNA damage enter mitosis, resulting in DNA fragmentation and micronuclei formation that trigger innate immunity [60,92,93]. Furthermore, the inhibition of the ATR effector kinase CHK1 has been observed to abrogate the G2/M checkpoint after irradiation, resulting in the production of micronuclei and the activation of the type I IFN pathway in cancer cells [94]. Additionally, combined radiotherapy and the CHK1 inhibitor AZD7762 in mice increased CD8+ T-cell infiltration and reduced the tumor volume compared to individual treatments with these agents [94]. Furthermore, ATR inhibition may enhance tumor immunogenicity by reducing the expression of programmed cell death 1 ligand 1 (PD-L1) in irradiated cancer cells [89,95,96]. Also, preclinical studies have indicated that cells that experience high replication stress may be selectively eliminated by ATR inhibition [97]. Indeed, the researchers observed that as the level of single-stranded DNA increased, a greater proportion of cells treated with ATR inhibitors underwent mitotic catastrophe. This finding suggests that the degree of replication stress and the extent of ATR inhibitor-induced single-stranded DNA could potentially predict sensitivity to ATR inhibition.

Lately, it has been shown that except for the induction of immune response through canonical cGAS/STING signaling, the combination of irradiation therapy and ATR inhibition can also activate non-canonical STING pathway [98]. As a result, a more robust immune activation was achieved, leading to increased type I interferon-related gene expression and T cell infiltration, turning the “cold” tumor microenvironment into “hot” and, thus, restoring sensitivity to PD-L1 immunotherapy [98].

Taken together, DDR-targeted therapies, including the inhibition of the ATR kinase, have the potential to increase the antitumor immune response through various mechanisms, including the augmentation of antigenicity, the promotion of genomic instability in tumor cells, the activation of cytosolic immunity, as well as the modulation of different components that influence the interaction between tumor and immune cells [99].

## 4. The ATR Pathway as a Therapeutic Target

Chemotherapy resistance is a common challenge in the treatment of cancer, as the activation of a functional DDR can lead to cell cycle arrest and prolonged DNA repair [6]. Blocking the ATR pathway can reverse this state and enhance the cytotoxicity of genotoxic drugs by abrogating the cell cycle checkpoint [23,100].

### 4.1. ATR Inhibition and Synthetic Lethality

A large-scale screening of in vitro and in vivo preclinical models of colorectal cancer has indicated that DDR inhibitors, in general, and ATR inhibitors specifically, are strong candidates for immunotherapy alternatives and has also suggested various response-predictive biomarkers for ATR inhibition, such as ATM protein loss [101]. Interestingly, recent data confirmed preclinical findings that the inhibition of ATR can lead to synthetic lethality in ATM-deficient malignancies. In fact, a study in ATM-deficient/p53-null cancer cells showed that ATR inhibition with VE-821 resulted in increased cytotoxicity after treatment with a variety of genotoxic agents, including platinum-based drugs, radiation, antimetabolites (gemcitabine), and topoisomerase inhibitors (camptothecin and etoposide). Importantly, VE-821 demonstrated a synergistic effect in tumor cells but not in normal cells [102]. Another study has also presented synergy between cisplatin and the ATR inhibitor ceralasertib (AZD6738) in ATM-deficient NSCLC (non-small cell lung cancer) cells [103]. Together, these data suggest that inhibition of ATR can lead to synthetic lethality in ATM-deficient/p53-null cancer cells that depend on alternative pathways to repair DSBs [104]. Strikingly, combining ceralasertib with cisplatin resulted in an enhanced cytotoxic effect even in ATM-proficient cell lines [105]. Of note, although previous studies have shown that ATM facilitates fork stabilization and maintains DNA replication [106] in ATM-proficient tumors, the ATR pathway also plays the most important role in replication stress management. For example, tumors expressing oncogenes (e.g., Ras, Myc), which are known to induce high replication stress [107], exhibited a strong response to ATR inhibition even without additional genotoxic treatment [108,109,110,111]. In fact, ATR appears to be crucial for the survival of those tumors, rendering ATR inhibition monotherapy a potential anticancer treatment [13,35].

Another striking example of induced synthetic lethality and antitumor immunity after ATR inhibition has been recently reported regarding Mismatch Repair (MMR) deficient cancer cells [112]. Also, in tumors with high levels of microsatellite instability (MSI-H) that are characterized by decreased levels of WRN helicase, it has been shown that ATR inhibition may potentiate tumor cell death [113]. MSI-H tumors may also present mutations in the ARID1A chromatin remodeling protein that plays a substantial role in DNA repair [114]. ARID1A loss of function is quite common, mostly among gynecological cancers, and renders the ATR pathway indispensable for ARID1A-deficient cells, as demonstrated in vitro and ex vivo in colorectal cancer (CRC) cells [114,115]. Mechanistically, ARID1A-deficient cells are characterized by loss of the G2/M cell cycle checkpoint and impaired homologous recombination. When treated with ATR inhibitors, genomic instability is induced, leading to cell death [114,115,116].

Taken together, the functionality of specific factors could be exploited as a predictive biomarker of ATR-blockade response, and, in parallel, combined perturbation of these proteins could lead to a synthetic lethality effect.

### 4.2. ATR Inhibitors Synergy with Other Anti-Tumor Therapies

ATR inhibitor AZD6738 has been proven to synergize with chemotherapy agents like cisplatin in various solid tumor preclinical models, resulting in augmented antitumor activity [105,117]. Likewise, berzosertib (VE-822, VX-970, M6620) has been found to increase cell death both in cell lines and in patient-derived primary lung xenografts after cisplatin treatment while also exhibiting a strong effect in tumor growth arrest in NSCLC models [118,119]. Other chemotherapy drugs may also be combined with ATR inhibition. Indeed, a recent study has shown synergism of the ATR inhibitor AZD6738 with the topoisomerase I inhibitor belotecan in ovarian cancer models [120], while combination with the antimetabolite gemcitabine in pancreatic models has been shown to instigate high replication stress leading to increased cell death and tumor shrinkage [121].

Recently, it has also been reported that AZD6738 can result in augmented cytotoxicity in vitro and tumor regression in vivo when combined with Trastuzumab Deruxtecan (T-DXd), an anti-HER2 antibody-topoisomerase I inhibitor hybrid [122], as well as improve the effectiveness of PI3K inhibitors, probably by DSBs-induced apoptosis as shown in in vitro and in vivo preclinical models of breast cancer [123].

An intriguing idea has led to testing the combination of ATR inhibition with poly (ADP-ribose) polymerase (PARP) inhibitors. PARP is an essential protein for multiple DDR pathways, and several inhibitors, such as olaparib, have been synthesized and are currently used in clinical practice. Olaparib induces DNA damage and activates BRCA1/2-dependent homologous recombination. Thus, it is used to cause synthetic lethality in BRCA1/2-deficient cancers or with synchronous administration of HR-blocking agents [124]. Accumulating data show that the ATR inhibitor AZD6738 synergizes with olaparib to overcome resistance and achieve induced cytotoxicity in ATM-deficient tumors and/or tumors with impaired HR repair [125,126]. However, it has also been proved that AZD6738 combined with olaparib and radiotherapy can benefit therapeutically even HR-proficient tumors through “PARP trapping” and the formation of PARP-DNA complexes that impede DNA replication [127].

A recent study underlined the significance of ATR inhibition scheduling during therapy [128]. The authors reported that to achieve increased cytotoxic T cells in the tumor-draining lymph node (DLN), radiation therapy or immune checkpoint inhibition must be followed by a short ATR inhibition rather than a prolonged one.

Interestingly, the contribution of ATM and ATR to the killing effect of the DNA-methylating drug temozolomide was investigated in several cell lines [129,130]. The authors reported that the knockdown of ATM and ATR increased cell killing of glioblastoma and melanoma cells with a more significant effect in the ATR knockdown cells, suggesting that the combination of temozolomide with an ATR inhibitor might be a promising approach to fight temozolomide resistance.

### 4.3. ATR Inhibition in Clinical Studies

Several clinical trials of ATR inhibitors are ongoing and promise to radically alter the treatment landscape in a variety of solid tumors, either as monotherapies or in combinational treatments. As we anticipate the results of the next phase trials in the upcoming years, we briefly present some of the most encouraging data from the clinic. One of the agents further along in clinical development is ceralasertib (AZD6738), with several phase 2 trials having completed recruitment and reporting clinical outcomes. Relevant clinical trials of ceralasertib in patients with solid tumors are listed in Table 1.

#### 4.3.1. Breast Cancer

A phase I trial of ceralasertib and olaparib included patients with pretreated, HRR-wild type metastatic triple-negative breast cancer (mTNBC) (*n* = 25) or BRCA-mutated HER2-negative metastatic breast cancer (*n* = 37). In the first cohort, no responses were observed, with a median progression-free survival (PFS) of 3.1 months (80% confidence interval [CI]: 2.0–3.9 months). In the BRCA-mutated cohort, the overall response rate (ORR) was 35%, with a median PFS of 7.7 months (80% CI: 5.8–11.4 months) [131]. The results of this study were further evaluated in VIOLETTE (NCT03330847), a randomized phase 2 trial evaluating the combination of ceralasertib and olaparib in comparison to olaparib monotherapy or the combination of olaparib and adavosertib, a WEE1 inhibitor, in patients with pretreated mTNBC. In this study (*n* = 273 patients), the combination of ceralasertib and olaparib did not improve PFS over olaparib monotherapy (7.3 vs. 7.4 months, hazard ratio [HR]: 1.02, 90% CI: 0.63–1.66, *p* = 0.94). Interestingly, while response rates were similar between the combination therapy and olaparib monotherapy (50% vs. 44%), the ORR with the combination therapy was higher in patients without HRR gene mutations (15% vs. 4%, odds ratio: 4.45; 90% CI: 1.30–21.20, *p* = 0.04) [132]. In the plasmaMATCH trial, which included patients with pretreated mTNBC (*n* = 70), the ORR was 17.1% (95% CI: 10.4–25.5%), with a median PFS of 4.3 months. Responses were observed in patients without BRCA1/2 mutations who had functional HR deficiency (HRD) by RAD51 foci [133]. This may account for the responses observed in patients without HRR gene mutations in the VIOLETTE study, and this subset of patients requires further evaluation in future clinical trials.

The combination of ceralasertib and olaparib, among other combinations, is being evaluated in mTNBC in another phase 2 trial that is actively recruiting patients (NCT03801369). Other than mTNBC, this combination is also being evaluated in HER2-negative, germline BRCA mutated advanced or metastatic breast cancer patients pre-treated with PARP inhibitors (NCT04090567). Trials evaluating other drug combinations include ATRiBRAVE (NCT05582538), an open-label phase 2 trial of mTNBC patients who have experienced disease progression after locoregional therapy that included chemotherapy and immunotherapy. In this trial, patients will receive a priming therapy by ceralasertib followed by the combination of paclitaxel and durvalumab, aiming to restore sensitivity to immunotherapy.

#### 4.3.2. Lung Cancer

The HUDSON trial (NCT03334617) is an open-label, biomarker-directed trial for patients with metastatic NSCLC (mNSCLC) after progression on chemotherapy and immunotherapy. In this trial, the combination of durvalumab with ceralasertib demonstrated early signals of efficacy in patients with ATM alterations (*n* = 18 patients, 6-month overall survival [OS]: 100%, 6-month PFS: 61.2%, ORR: 13.3%) and in unselected patients (*n* = 20 patients, 6-month OS: 74.8%, 6-month PFS: 53.8%, ORR: 11.1%) [134]. As a result, a randomized phase 2 trial (NCT03833440) and a randomized phase 3 trial (NCT05450692) are ongoing, which compare the combination of durvalumab and ceralasertib in mNSCLC patients who have progressed on chemotherapy and immunotherapy with the standard of care in this indication (docetaxel) and may be practice-changing in this setting. In another open-label, biomarker-directed clinical trial, the National Lung Matrix Trial (NLMT), which included pretreated, KRAS-mutated or KRAS-wild type mNSCLC patients that had received prior immunotherapy, outcomes were numerically higher in patients with KRAS mutations (ORR: 13.8% vs. 4.8%, mPFS 5.95 vs. 3.9 months, mOS 30.9 vs. 13.2 months).

In extensive-stage small cell lung cancer (ES-SCLC), a small phase 2 trial is ongoing in the first-line setting that evaluates the efficacy of maintenance therapy with ceralasertib plus durvalumab after four cycles of induction therapy with platinum-etoposide-based chemotherapy and durvalumab (NCT04699838). Results have been reported from an open-label phase 2 trial that included 21 patients with platinum-refractory ES-SCLC that received ceralacertib plus olaparib (NCT02937818), the ORR was 4.8%, with a 12-week disease control rate of 38.1%. Interestingly, despite the disappointing response rate, the median OS for patients who received the combination therapy was 7.56 months, which is in line with approved therapies for this indication. Similar results were seen in SUKSES, a phase 2 umbrella trial that included patients with refractory ES-SCLC who received the combination therapy. In this study, the ORR was 3.8%, the median PFS was 2.75 months (95% CI: 1.77–5.44 months), and the median OS was 7.18 months (95% CI: 5.97–10.79 months) [135].

#### 4.3.3. Gynecological Cancers

The combination of ceralasertib and olaparib has shown early clinical activity in patients with advanced, high-grade serous ovarian cancer (HGSOC) that have progressed after treatment with a PARP inhibitor. In OLAPCO, a basket trial of olaparib combinations in heavily pre-treated patients, responses or prolonged disease stabilization with ceralasertib plus olaparib were observed in patients with ATM mutations and those who had received prior treatment with a PARP inhibitor [136]. In CAPRI, an open-label phase 2 trial of the same combination, promising clinical activity was seen in patients with HGSOC enrolled immediately after progression on a PARP inhibitor (*n* = 13), with an ORR of 46% [137]. No responses were found in patients with platinum-resistant disease (mPFS was 4.2 months overall (90% CI: 3.5–8.2 months) [138].

The combination of ceralasertib and olaparib has also been evaluated in rare gynecological cancers in the open-label phase 2 ATARI trial (*n* = 78). In this study, outcomes were similar in patients with clear cell histology with or without AT-rich interactive domain-containing protein 1A (ARID1A) loss (ORR: 14% vs. 14%, median PFS: 3.6 vs. 3.5 months), while the clinical activity of the combination therapy may be higher in those with non-clear cell histologies (ORR: 24%, median PFS: 5.6 months) [139].

#### 4.3.4. Other Solid Tumors

In PATRIOT, the first-in-human trial of ceralasertib in patients with advanced solid tumors, a subset of patients with ARID1A deficiency derived greater benefit from treatment [140]. Furthermore, an open-label phase 2 trial in patients with advanced solid tumors also included a cohort of ARID1A-deficient tumors. In this cohort, durable complete responses were achieved in 2/10 patients for an ORR of 20% [141]. ARID1A deficiency represents a promising target for ATR inhibition that warrants future evaluation.

The combination of ceralasertib plus durvalumab has been evaluated in various solid tumors. In an open-label phase 2 trial of the combination therapy in patients with pretreated advanced gastric cancer (*n* = 31), the ORR was 22.6% (95% CI: 9.6–41.1%), the median PFS 3.0 months (95% CI: 2.1–3.9 months), and the median OS 6.7 months (95% CI: 3.8–9.6 months). The benefit was limited to patients with ATM deficiency or a mutational signature attributable to HRD (median PFS: 5.60 vs. 1.65 months, HR: 0.13, 95% CI: 0.045–0.39, *p* < 0.001) [142]. This study also included a cohort of patients with melanoma who had progressed on treatment with a PD-1 inhibitor (*n* = 30). In this cohort, the ORR was 31.0%, the median PFS was 7.1 months (95% CI: 3.6–10.6 months), and the median OS was 14.2 months (95% CI: 9.3–19.1 months) [143].

In addition to PARP and immune checkpoint inhibitors, ceralasertib is also evaluated in combination with chemotherapy. In a phase I trial of ceralasertib plus weekly paclitaxel in patients with advanced solid tumors, the combination was safe and showed preliminary signs of efficacy, with an ORR of 25.4%, including one complete response in a patient with melanoma [144]. A phase I trial of ceralasertib plus gemcitabine in patients with advanced solid tumors is ongoing (NCT03669601).

## 5. Conclusions

Taken together, data present in this report demonstrate that the inhibition of the ATR kinase can modify the DNA damage response network and the immune system. These results potentially offer a new approach to improving the effectiveness of anticancer therapy using combinations of an ATR inhibitor with genotoxic drugs and/or immunomodulators, with promising early signals of efficacy in lung cancer, melanoma, and gastric cancer. Given the limited responses seen with single-agent use of ATR inhibitors, future clinical trials should focus on further evaluation of combination strategies and on discovering novel predictive biomarkers of response.

## Figures and Tables

**Table 1:** Clinical trials of Ceralasertib (AZD6738) in patients with solid tumors.

NCT Number	Study Status	Conditions	Interventions	Primary Outcome	Phase	Enrollment	Completion Date
NCT03330847	Active, not recruiting	mTNBC	Ceralasertib + Olaparib	PFS	2	273	September 2024
NCT03801369	Recruiting	mTNBC	Ceralasertib + Olaparib	ORR	2	132	December 2027
NCT03740893	Recruiting	Operable TNBC	Ceralasertib	Biomarker	2	81	December 2025
NCT03182634	Completed	mBC	Ceralasertib + Olaparib	ORR	2	70	November 2023
NCT04090567	Recruiting	HER2−, BRCA+ mBC	Ceralasertib + Olaparib	ORR	2	60	March 2025
NCT05582538	Recruiting	mTNBC	Ceralasertib followed by Durvalumab/nab-Paclitaxel	PFS	2	37	November 2025
NCT05450692	Recruiting	mNSCLC	Ceralasertib + Durvalumab	OS	3	580	May 2025
NCT03334617	Active, not recruiting	mNSCLC	Ceralasertib, Ceralasertib + Durvalumab	12-week ORR	2	531	September 2024
NCT02664935	Active, not recruiting	mNSCLC	Ceralasertib + Durvalumab	ORR, PFS, 24-week DCR	2	423	September 2023
NCT03833440	Recruiting	mNSCLC	Ceralasertib + Durvalumab	12-week DCR	2	120	February 2024
NCT05941897	Recruiting	mNSCLC	Ceralasertib + Durvalumab	ORR	2	38	June 2025
NCT02937818	Active, not recruiting	ES-SCLC	Ceralasertib + Olaparib	ORR	2	72	December 2023
NCT04361825	Active, not recruiting	ES-SCLC	Ceralasertib + Durvalumab	ORR	2	45	December 2023
NCT04699838	Recruiting	ES-SCLC	Platinum-Etoposide-Durvalumab + maintenance Ceralasertib/Durvalumab	PFS	2	30	May 2024
NCT03428607	Completed	ES-SCLC	Ceralasertib + Olaparib	ORR	2	26	January 2021
NCT03579316	Recruiting	Ovarian Cancer	Ceralasertib + Olaparib	ORR	2	104	December 2024
NCT04239014	Withdrawn	Ovarian Cancer	Ceralasertib + Olaparib	PFS	2	0	January 2021
NCT04065269	Active, not recruiting	Gynaecological Cancers	Ceralasertib + Olaparib	ORR	2	168	March 2023
NCT05061134	Active, not recruiting	Melanoma	Ceralasertib, Ceralasertib + Durvalumab	ORR	2	186	April 2024
NCT03780608	Active, not recruiting	Melanoma, Gastric cancer	Ceralasertib + Durvalumab	ORR	2	61	December 2023
NCT04298021	Active, not recruiting	Biliary Tract Cancer	Ceralasertib + Durvalumab, Ceralasertib + Olaparib	DCR	2	74	December 2024
NCT04298008	Recruiting	Biliary Tract Cancer	Ceralasertib + Durvalumab	DCR	2	26	December 2024
NCT04417062	Recruiting	Osteosarcoma	Ceralasertib + Olaparib	4-month EFS	2	63	June 2025
NCT03787680	Active, not recruiting	Prostate Cancer	Ceralasertib + Olaparib	ORR	2	49	January 2027
NCT03022409	Completed	HNSCC	Ceralasertib	Biomarker	1	21	January 2021
NCT04704661	Recruiting	HER2+ GEJ/CRC	Ceralasertib + T-DXd	Toxicity	1	15	March 2026
NCT02264678	Recruiting	Advanced Solid Tumors	Ceralasertib + Olaparib	Toxicity	1/2	466	July 2026
NCT03682289	Recruiting	Advanced Solid Tumors	Ceralasertib, Ceralasertib + Olaparib	ORR	2	89	July 2025
NCT02223923	Active, not recruiting	Advanced Solid Tumors	Ceralasertib	MTD	1	87	December 2023
NCT02576444	Terminated	Advanced Solid Tumors	Ceralasertib + Olaparib	ORR	2	67	November 2019
NCT02630199	Completed	Advanced Solid Tumors	Ceralasertib + Paclitaxel	Toxicity, MTD	1	65	April 2021
NCT03669601	Recruiting	Advanced Solid Tumors	Ceralasertib + Gemcitabine	DLT	1	55	September 2024
NCT04564027	Active, not recruiting	Advanced Solid Tumors	Ceralasertib	ORR	2	54	February 2024
NCT05514132	Active, not recruiting	Advanced Solid Tumors	Ceralasertib + Olaparib	DLT	1	14	April 2025
NCT05469919	Active, not recruiting	Advanced Solid Tumors	Ceralasertib	DLT	1	12	December 2024
NCT03878095	Suspended	IDH1/2 mut Advanced Solid Tumors	Ceralasertib + Olaparib	ORR	2	50	March 2024
NCT03330847	Active, not recruiting	mTNBC	Ceralasertib + Olaparib	PFS	2	273	September 2024
NCT03801369	Recruiting	mTNBC	Ceralasertib + Olaparib	ORR	2	132	December 2027
NCT03740893	Recruiting	Operable TNBC	Ceralasertib	Biomarker	2	81	December 2025
NCT03182634	Completed	mBC	Ceralasertib + Olaparib	ORR	2	70	November 2023
NCT04090567	Recruiting	HER2−, BRCA+ mBC	Ceralasertib + Olaparib	ORR	2	60	March 2025
NCT05582538	Recruiting	mTNBC	Ceralasertib followed by Durvalumab/nab-Paclitaxel	PFS	2	37	November 2025
NCT05450692	Recruiting	mNSCLC	Ceralasertib + Durvalumab	OS	3	580	May 2025
NCT03334617	Active, not recruiting	mNSCLC	Ceralasertib, Ceralasertib + Durvalumab	12-week ORR	2	531	September 2024

Abbreviations: CRC, colorectal carcinoma; DCR, disease control rate; DLT, dose-limiting toxicity; EFS, event-free survival; ES-SCLC, extensive-stage small cell lung cancer; GEJ, gastroesophageal junction; HNSCC, head and neck squamous cell carcinoma; IDH1/2 mut, isocitrate dehydrogenase 1/2-mutated; mBC, metastatic breast cancer; mNSCLC, metastatic non-small cell lung cancer; MTD, maximum tolerated dose; mTNBC, metastatic triple negative breast cancer; ORR, overall response rate; OS, overall survival; PFS, progression-free survival; T-DXd, Trastuzumab Deruxtecan; TNBC, triple negative breast cancer.

## Data Availability

The data presented in this study are openly available in the reference section.
